# Variola, Vaccinia, Varioloid, and Varicella

**Published:** 1847-09

**Authors:** 


*Variola, Vaccinia, Varioloid, and Varicella.*—Dr. Koesch, the author of any essay published under the above title, conconcludes:

1. That cow-pock is nothing more than small-pox, transmitted to the cow by contact.

2. That persons who have been effectually vaccinated may, in some rare instances, contract dangerous small-pox.

3. That small-pox after vaccination is, in the great majority of cases, of trifling severity.

4. That the rarity and mildness of small-pox are in proportion to the recency of the vaccination.

5. That small-pox seldom appears after the age of thirty, but is not always less severe when it does so.

6. That the majority of the vaccinated are entirely exempt from small-pox, even though exposed to contagion.

7. The identity of variola and varioloid is demonstrated by their phenomena, development, and by the results of contagion or inoculation.

8. That varicella is in nowise connected with variola, but is a perfectly distinct disease.

9. That vaccination is the only mode of exterminating small-pox.—*Ibid.*

